# Implementation and effectiveness of continuous kangaroo mother care: a participatory action research protocol

**DOI:** 10.1186/s13006-021-00367-3

**Published:** 2021-03-08

**Authors:** Marzieh Mohammadi, Anne-Marie Bergh, Mohammad Heidarzadeh, Mahammadbagher Hosseini, Niloufar Sattarzadeh Jahdi, Leila Valizadeh, Behzad Sarvaran, Sevil Hakimi

**Affiliations:** 1 Student Research Center Committee, Tabriz University of Medical Science, Tabriz, Iran; 2 SAMRC Unit for Maternal and Infant Health Care Strategies and Research Centre for Maternal, Fetal, Newborn and Child Health, University of Pretoria, Pretoria, South Africa; 3 Pediatric health research center, Tabriz University of Medical Science, Tabriz, Iran; 4 Department of Midwifery, School of Nursing and Midwifery, Tabriz University of Medical Science, Tabriz, Iran; 5 Department of Pediatric Nursing, Tabriz University of Medical Science, Tabriz, Iran; 6 Taleghani Training Hospital, Tabriz University of Medical Science, Tabriz, Iran; 7 Department of midwifery, School of Nursing and midwifery, Research center of psychiatry and behavioural science, Tabriz University of Medical Science, Tabriz, Iran

**Keywords:** Continuous kangaroo mother care, Health services research, Premature infant, Health plan implementation

## Abstract

**Background:**

The efficacy of continuous kangaroo mother care (C-KMC) in reducing neonatal mortality and morbidity among low birthweight and premature infants has been confirmed. Despite the recommendations of the World Health Organization, UNICEF, and the Ministry of Health of Iran to use C-KMC for eligible hospitalized neonates, this type of care is not performed due to implementation problems. This protocol aims to describe the design, implementation, and assessment of C-KMC in one tertiary hospital by means of participatory action research.

**Methods:**

The objective of this study is to design and implement a C-KMC program for neonates that will be performed in two phases using a stages-of-change model. The first phase will be conducted in three consecutive activities of designing, implementing, and assessing the introduction of C-KMC. The second phase of the study has a before-and-after design to assess the effectiveness of C-KMC by comparing the length of preterm neonates’ stay in hospital and exclusive breastfeeding at discharge before and after implementing C-KMC.

**Discussion:**

KMC is an important component of neonatal developmental care as part of family-centered care. Applying this type of care requires creating appropriate strategies, budget allocation, and clear and coordinated planning at different levels of the health system. The stages-of-change model is one of the appropriate approaches to the implementation of C-KMC.

## Background

An estimated 2.5 million newborns die every year, of which the vast majority of deaths occurs in low- and lower-middle-income countries [1]. Premature birth is the leading cause of neonatal deaths around the world, accounting for 47% of deaths in children below 5 years of age in 2018 [2]. In addition to premature infants being at greater risk for neonatal mortality, these infants suffer more long-term health problems, physical disabilities, and lifelong mental disabilities than term infants [3]. Providing care for premature newborns imposes a heavy burden on healthcare [4, 5] and effective interventions require high technology, skilled staff, and an efficient care system in addition to high costs [6].

In line with the goals of sustainable development to reduce infant mortality in low-income and middle-income countries, we need low-cost care with high effectiveness [7].

KMC is a cost effective complementary method of caring for stable low birthweight (LBW) and preterm neonates [8, 9] that can contribute to improving the quality of care, as it ensures constant temperature regulation and improved survival of these infants. C-KMC is one of the 10 recommendations of the World Health Organization (WHO) for providing care for premature neonates [7]. Evidence-based studies have shown the efficacy of KMC to reduce mortality and morbidity in preterm neonates, prevent hypothermia and infection, improve maternal-infant attachment and increase the duration of exclusive breastfeeding [8–14].

KMC can be provided intermittently and continuously. Nyqvist et al. describe the difference as follows: “The hallmark of KMC is the kangaroo position: the infant is cared for skin-to-skin vertically between the mother’s breasts and below her clothes, 24 h / day, with father / substitute(s) participating as KMC providers. Intermittent KMC (for short periods once or a few times per day, for a variable number of days) is commonly employed in high-tech neonatal intensive care units.” [15].

Studies have shown that the duration of KMC can be increased by improving facilities [16]. The longer the care duration is, the greater the benefits obtained from it are and the better consequences of physical, mental and emotional development are for the premature infant who needs this care [6, 15].

Despite the recommendations of the WHO and UNICEF, on the use of C-KMC for eligible hospitalized neonates, this type of care is not implemented because of various barriers such as lack of physicians’` and nurses` preferences as well as limitation in appropriate place for carry out C-KMC. All cross-sectional studies conducted in Iran so far have focused on intermittent KMC [6, 17, 18] and the number of hours of KMC per day was below the recommended average for intermittent KMC [19].

### Study aim

This protocol describes a proposed study aimed at designing a C-KMC program for neonates and implementing and evaluating this program and the effect of C-KMC in a teaching hospital in North West Iran. The hospital had nearly 6500 deliveries in 2018, with 8% of neonates being preterm. There is a level III neonatal intensive care unit (NICU) with 25 beds and a neonatal ward with 15 beds.

At present, infants admitted to the neonatal ward or NICU receive 1 to 2 hours of KMC per shift at this hospital. According to the protocol, the minimum duration of this care is 1 hour. Care will continue after 1 hour as long as the mother has the ability and patience to do it. Typically, mothers can do this care for more than 2 hours in a shift. Consequently, each baby receives care for three to 6 hours (according to the mother’s ability) until now and before performing this plan.

The specific objectives of this study are to:

1. Identify the barriers and problems related to the introduction of C-KMC
2. Implement C-KMC according to the principles of action research
3. Evaluate progress with C-KMC implementation
4. Compare exclusive breastfeeding rates at the time of hospital discharge for a period before and after introducing C-KMC
5. Compare the duration of hospitalization of neonates before and after the introduction of C-KMC

### Study design

This study uses a mix- method design, which will be conducted in two phases. The first phase will use a participatory action research approach in three consecutive activities (design, implementation and evaluation) as part of the introduction of C-KMC. The second phase of the study is a quantitative before-and-after study aimed at assessing the effectiveness of C-KMC. Table 1 gives an overview of the study design.

**Table 1:** Overview of the study design

	Phase 1: Tracking C-KMC implementation	Phase 2: Measuring effectiveness of C-KMC
Intervention	Continuous kangaroo mother care	
Study design	Participatory action research	Before-after comparison of non-C-KMC and C-KMC neonates
Aim	To implement continuous KMC in the study setting and identify barriers / challenges to the implementation of C-KMC	To investigate the effectiveness of C-KMC
Hypothesis	Not applicable	Continuous KMC: 1. reduces the length of hospital stay of preterm neonates / neonates <2500g 2. increases the rate of exclusive breastfeeding at discharge
Study setting	General hospital in North West Iran, level III NICU^a^	
Participants	• Mothers with preterm neonates • Nurses and midwives • Managers and health policy makers	• Preterm neonates
Sampling	• Purposive sampling • Sample size: until data saturation is reached	• Consecutive sample (before and after C-KMC implementation) • Sample size: 208 = 104 per group
Data collection tools	1. Interview guide (for focus group discussions and individual interviews) 2. Standardized progress-monitoring tool: • Observations of service provision, care and records • Interviews with health care providers (nurses, doctors, etc.)	Data collection sheet (questionnaire): • Length of hospitalization (total and length of days in C-KMC) • Feeding method(s) at discharge • Other characteristics (e.g. gestational age and sex) – see Table 2 for details
Data collection process	• Focus group discussions (health care providers and managers) and individual interviews (mothers) • Application of the progress-monitoring tool	Completion of the data collection sheet using data from the neonates’ medical file
Data analysis	• Qualitative content analysis • Software: MAXQDA10	• Data with normal distribution: paired t-test; Pearson correlation coefficient; ANOVA • Paired nominal data: McNemar’s test • Software: SPSS version 24
Ethical considerations	Written consent from participants before interviews and focus groups	Consent from parents or hospital management to use data
	Approval by the Ethics Committee of Tabriz University of Medical Sciences, Tabriz, Iran	

^a^*NICU level III* Neonatal intensive care unit that is capable of caring for neonates <32 weeks or <1500 g with professional personnel including neonatal nurses who are available 24 hours a day, *ANOVA* analysis of variance, *SPSS* Statistical Package for Social Sciences, *C-KMC* contionuse kangaroo mother care

### First study phase: tracking implementation

**Fig 1:**
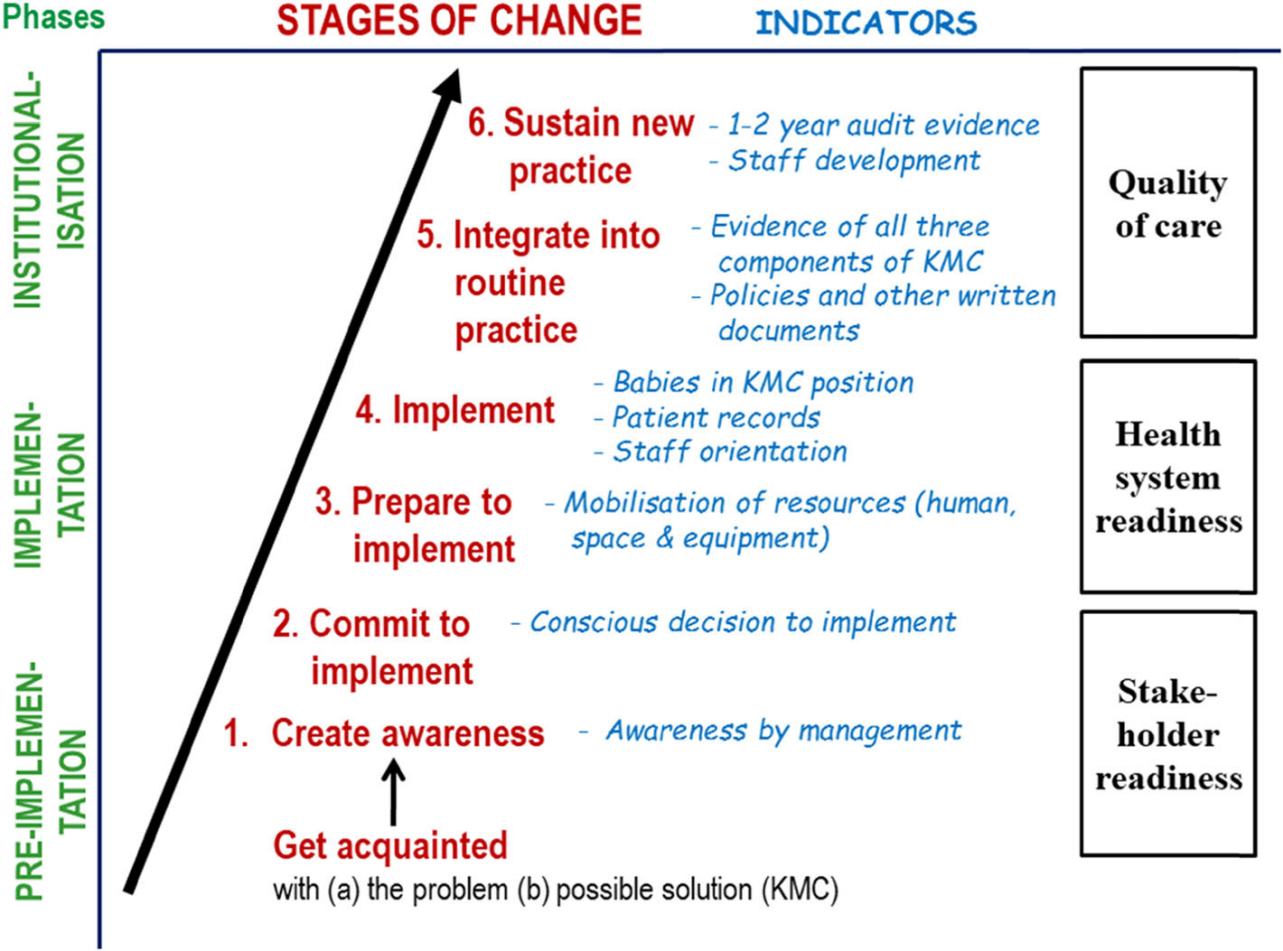
Stages-of-change model for implementing C-KMC

The participatory action research in this study is based on the approach of the stages-of-change model developed by Bergh et al. [20]. This model is a useful model for introducing C-KMCs in hospitals and is almost consistent with McNiff’s research action [21]. The stages-of-change model includes three main change phases, namely pre-implementation (readiness of stakeholders), implementation (readiness of system) and institutionalization (quality of care). Each phase consists of two steps or stages. Figure 1 illustrates this model for implementing C-KMC. The research group will use the results of the first study phase to inform the authorities about current care and necessary adaptations to improve the care with a view to get their commitment for the further administration and expansion of C-KMC.

### Second study phase: measuring effectiveness of C-KMC

In this study phase, the effectiveness of C-KMC implementation will be assessed by means of two measures in a before-after comparison: length of eligible neonates’ stay in hospital and exclusive breastfeeding rate at the time of hospital discharge.

## Methods

### Description of continuous kangaroo mother care as intervention

The following steps will be taken to initiate the introduction of C-KMC:

- Identifying the roles of individuals and training them in the necessary issues.
- Compiling instructions and forms in the process of getting ready for C-KMC administration.
- Preparing the physical space for C-KMC and ablution facilities, as well as acquiring furniture (e.g. beds and lockers or wardrobes) and organizing catering services to provide meals for mothers.

Once the KMC ward is prepared, the implementation of C-KMC will include the following:

- The pediatrician will identify infants eligible for C-KMC.
- Mothers will be counselled on how to do C-KMC including skin-to-skin care, breastfeeding and the requirements for follow-up care after discharge from the hospital.
- Mothers will be allowed to freely move, watch TV, study and do other activities of interest.

As part of the implementation, the research group will identify the strengths and weaknesses of the program and make the necessary adjustments and revisions to establish C-KMC as routine practice in the KMC ward.

### Sampling

**Phase 1** of the study will investigate the challenges and barriers to the introduction of C-KMC in line with the first stage of the stages-of-change model. Participants at this stage will include mothers with preterm neonates, nurses, midwives, managers, and health policy makers who are associated with the neonatal ward where C-KMC is to be implemented. They will be selected through purposeful sampling to participate in focus group discussions and individual interviews. Sampling will continue until the data is saturated, i.e. no new information is obtained [22].

Participants for the focus groups will be healthcare providers and managers allocated to homogeneous groups (e.g. nurses or doctors only) and heterogeneous groups (e.g. health officials, hospital directors, nursing services managers, nurses and doctors in the same group). This will enable the evaluation of challenges, obstacles and opportunities for improvement from various perspectives. Mothers will be interviewed individually with a view to identifying their special needs.

**Phase 2** of the study will include two groups of premature neonates as study participants: those that received C-KMC (intervention) and those that did not receive C-KMC (control). The sample size in this part of the study was determined according to the criteria used in the study conducted by Mishra et al. [23]. For the days of stay in hospital (m1 = 14, SD1 = 7, m2 = 12, and SD2 = 6), α = 0.05 β = 0.2, the sample size was determined to be 102 people in each group. The total sample size was estimated to be 204 participants. The ratio of the intervention to control group will be 1 to 1.

Inclusion criteria for preterm neonates in both groups are birthweight ranged between 1200 and 2500 g, stable condition, spontaneous breathing (no need for CPAP / ventilator), no oxygen therapy, ability to feed by tube or directly from the breast, and absence of maternal disease (e.g. active tuberculosis and severe maternal depression and anxiety). All infants included in this phase of the study would have received intermittent KMC in the NICU. For the intervention group, an additional inclusion criterion is pediatrician permission to initiate C-KMC.

Exclusion criteria include neonates with problems such as hypothermia (despite efforts to keep the baby warm), seizure, breathing problems (apnea, chest retraction, grunting, nasal flaring, and cyanosis), drowsiness (excessive sleep or reduced activity), and jaundice requiring phototherapy along with an unstable condition. In addition, maternal unwillingness to practice C-KMC will also be an exclusion criterion.

### Data collection and measurements

**Phase 1** (introduction of C-KMC) will include qualitative focus group and individual interviews that will be conducted with an interview guide with a few predetermined questions, followed by open-ended, in-depth exploratory questions relating to the barriers to C-KMC. Every interview will have a moderator and an administrator.

Information gathered in group discussions can be clarified and explored further by conducting individual interviews. In the focus groups information will be obtained by involving all members in the discussion and exploring proposed solutions for any barriers reported. The strengths and weaknesses of each solution will be discussed and any further barriers to implementation of the process.

Suggested questions will be:

1. How familiar were you with C-KMC?
2. How interested are you in C- KMC?
3. To what extent is performing C-KMC necessary in neonatal unit?
4. What are the most important barriers in launching C-KMC in your opinion?
5. How can these barriers be removed?

All interviews will be audio-recorded with the participants’ permission and will last between 45 min and 1 hour. Interviews will take place in a private room in the hospital.

The standardized tool developed from Bergh et al.’s [20] stages-of-change model will be used to monitor the implementation progress of C-KMC. This tool has 18 sections, of which some are completed by health personnel and some are completed as part of the observation of the unit and the C-KMC implementation process. The tool uses a scoring system with a maximum of 30 points. The score obtained by a hospital indicates the status of the hospital in terms of implementing C-KMC. Obtaining a score more than 10 indicates that the hospital is on track with implementing C-KMC. A score of more than 17 indicates that the hospital is on the track with providing routine C-KMC, whereas a score of more than 24 indicates that the care has been institutionalized and is moving towards the stage of sustaining practice [20].

**Phase 2** (evaluating the effectiveness of C-KMC) will employ a researcher-developed questionnaire (data collection sheet) as data collection tool. Possible items to be included in the questionnaire are listed in Table 2. The questionnaire will be sent to 10 pediatricians, nursing faculty members, midwives and nurses working in the neonatal intensive care unit to assess content validity. Their comments will be accommodated in the final version of the tool.

**Table 2:** Data to be captured in Phase 2 of the study

Focus areas	Specific information	
**Pregnancy history**	Pregnancy risk factors / complications	
	Delivery mode	
	Maternal age	
**Neonatal characteristics**	Gestational age	
	Sex	
	Medical needs at birth and in NICU	• Need for resuscitation • Need for respiratory support • Medical complications • Infection • Intervention therapies • Medication prescriptions
	Weight	• Birth weight (on admission to NICU/neonatal ward) • Weight on admission to C-KMC • Discharge weight
	Head circumference	• At birth • On admission to C-KMC • On discharge
	Body temperature	• On admission • During hospitalization in the NICU • During C-KMC • On discharge
**Operational characteristics**	Type of feeds – breast milk (tube/finger/direct), formula, mixed)	• In NICU • In C-KMC • At discharge
	Practice of C-KMC	• Intermittent KMC in NICU (Yes/No) • Mean duration of C-KMC (hours per day) • Maternal cooperation during C-KMC • Attendant to assist / substitute mother in doing C-KMC • Family support with and attitudes towards KMC
	Duration of hospitalization (days)	• In NICU • In C-KMC
**Discharge**	Discharge permission signed by doctor	
**Parental socio-demographic**	Mother’s educational level	
	Father’s educational level	
	Place of residence	
	Family income	

### Data analysis

#### Phase 1

Focus group and individual interviews will be transcribed verbatim and transcriptions will be read and re-read several times to identify meaningful units to be coded and from which themes will be extracted and categorized. MAXQDA10 software will be used for doing qualitative content analysis according to a conventional approach [24]. Four criteria: credibility, reliability, conformability, and transferability, will be applied to increase the trustworthiness of qualitative findings [25].

Data derived from the progress-monitoring tool will be analyzed quantitatively and qualitatively.

#### Phase 2

‘The quantitative cross-sectional data will be compared before and after the implementation of C-KMC to specify to what extent the change has been effective.’

Quantitative data obtained from the completed data collection sheet will be analyzed with SPSS version 24 software to generate descriptive and inferential statistics. Participants in the C-KMC and non-C-KMC groups will be matched for gestational age and sex. For data with a normal distribution, the paired t-test, Pearson correlation coefficient, and ANOVA will be used; for paired nominal data, McNemar’s test will be used.

## Discussion

According to the WHO recommendations, every neonate should receive the highest quality of care [26]. Studies have shown that the NICU faces the greatest number of care challenges, of which the provision of poor quality neonatal care is the most important challenge [27]. Nursing errors are an example of the dangers working in a NICU, where shortage of staff and high workload are common [28]. Family-centered care engages parents in providing care for premature neonates, reduces nurses’ workload and enhances parental confidence and competence in newborn care. WHO has considered KMC to be a part of family-centered care for sick and small infants [6].

KMC is an important component of neonatal developmental care that requires guiding and promoting clinical behavioral changes in medical staff [29].

In addition, KMC has many proven benefits. Studies have shown that KMC reduces neonatal crying, improves physiological conditions, and stabilizes cardiac and vascular status of neonates [30]. Skin-to-skin care reduces the cortisol levels in neonates and mitigates pain in these infants, making the painful neonatal processes more tolerable [31].

The longer the care duration is, the greater the benefits obtained [15, 32].

C-KMC improves neonatal development and reduces risk of growth retardation in the first 24 months. Its benefits can be observed even in adolescence and adulthood [18]. Applying this care requires the development of appropriate strategies, budget allocation and clear and coordinated planning at different levels of the health system. Employees’ lack of awareness, insufficient facilities and problems related to the mother, can interfere with the implementation of quality care. At all stages, problems, obstacles and achievements will be shared with participants and will be rethought.

Action research can be considered as a suitable approach to apply knowledge in practice and to fill the gap between performance and research in a clinical situation. Applying this approach, after understanding and recognizing the problem in context facilitates more informed decisions to be made to change the situation [33].

Action research leads to the transformation of the current situation into a more desirable situation, and action is the main focus in this research type. The purpose of this action is to improve affairs and make them effective [34]. This method is the best approach in applying new processes in the system and creating sustainability. Change is considered as a part of the research process and is done in the form of a repetitive cycle, consisting of stages to recognize the problem or situation that needs change, planning, action and then finding the results of the action for planning and next action [33, 35].

Applying C-KMC requires the development of appropriate strategies, budget allocation and clear and coordinated planning at different levels of the health system [36]. It seems that the use of a participatory action research approach would be useful in introducing C-KMC in the study hospital and the results could be used by health policy makers and planners to improve the quality of neonatal care.

## Data Availability

Not applicable.

## Abbreviations

ANOVA test: Analysis of variance test
C-KMC: Continues kangaroo mother care
CPAP: Continuous positive airway pressure
NICU: Neonatal intensive care unit
WHO: World Health Organization
